# Frailty assessment of older adults, first-time applicants of public home care service in Norway

**DOI:** 10.1080/02813432.2021.1880069

**Published:** 2021-02-08

**Authors:** Ingerid Laukli, Leiv Sandvik, Heidi Ormstad

**Affiliations:** a Faculty of Health and Social Sciences, University of South-Eastern Norway, Drammen, Norway; b Department of Activity and Rehabilitation, Drammen Municipality, Drammen, Norway; c Faculty of Health Sciences, Oslo Metropolitan University, Oslo, Norway

**Keywords:** Frailty, physical functional performance, primary health care, screening, walking speed

## Abstract

**Objective:**

Early detection of frailty is essential to prevent or delay disability. The most appropriate screening tool for frailty among home-dwelling older adults is under debate. The present study estimates the prevalence of frailty among older adults, first-time applicants of public home care service in Norway, and investigates the appropriateness of gait speed and Short Physical Performance Battery as screening-tools for frailty.

**Design and setting:**

We conducted a cross-sectional study of 116 older adults >65 years applying for public home care service for the first time. Frailty was assessed by an adapted version of the Fried Frailty Phenotype. The test accuracies of gait speed and Short Physical Performance Battery to detect frailty were calculated for a general population >70 years in Norway.

**Results:**

62.1% of the participants were frail, 29.3% were prefrail, and 8.6% were robust. Mean gait speed and Short Physical Performance Battery-scores were significantly lower in frail compared to prefrail individuals, and significantly lower in prefrail compared to robust individuals. The sensitivity and specificity of gait speed at a cut point of 0.8 m/s to detect physical frailty phenotype was 99% and 68%, respectively.

**Conclusions:**

The high prevalence of frailty in the present study indicates that screening for frailty should be considered at an earlier time point than when older adults apply for public home care service for the first time. Gait speed may be an appropriate screening tool for frailty in a general population >70 years in Norway.

**KEY POINTS**
- The prevalence of frailty among older adults, first-time applicants of public home care services in Norway is major.
- Screening for frailty should be considered before older adults apply for public home care service for the first time.
- Gait speed at a cut point at 0.8 m/s may be an appropriate screening tool for frailty in a general population >70 years in Norway.

## Backgrounds

Frailty is an identifiable decline in many physiological systems in late life, which results in an impaired response to minor stressors like acute illness or trauma, and is associated with an increased risk of adverse outcomes like falls, disability, delirium, hospitalization, and death [1]. There is evidence that preventing frailty can avoid many of the major negative health-related outcomes associated with ageing, contributing to healthy ageing [2]. Globally, it represents a severe public health challenge, an economical burden to the health care systems [3], reduced quality of life for individuals, and a burden to the next of kin [4,5]. The pooled frailty prevalence in community-dwelling older adults is reported to be 17.4% in middle-income countries versus 10.9% in high-income countries [6,7]. In Norway, the prevalence was 3.8% in a general population >70 years [8]. The prevalence of the intermediate state, prefrailty, was 49.3%, 41.6%, and 38.1% for middle-income countries, high-income countries, and Norway, respectively [6–8].

Among multiple diagnostic tools that define and measure frailty, the Frieds’ Frailty Phenotype (FFP) index is one of the most widely used [1]. The instrument is well validated with a good predictive value on adverse outcomes. According to Fried et al.(2001), frailty is defined by meeting three or more of the following criteria: unintentional weight loss, exhaustion, low physical activity, slowness, and weakness [9]. Prefrailty is defined by meeting one or two of the criteria and represents an intermediate increased risk to adverse outcomes. Diagnosing frailty using the FFP index or other diagnostic tools is time demanding and requires special instruments. It is therefore of interest to find quicker, low-cost screening tools to detect frailty [10,11]. There are multiple valid screening instruments for frailty [3,11]. Two tests, assessment of gait speed (GS) and the Short Physical Performance Battery (SPPB), are well known and frequently used in physical assessments of older adults in Norway [12]. The SPPB is originally a physical performance test that measures the physical components GS, standing balance, and repeated chair stands. It is quick, safe, and easy to administer. Each three parts give a score from null to four, then the maximum score is twelve. The test is validated to predict disability and all-cause mortality in community-dwelling older adults [13]. SPPB is also used as a diagnostic tool to detect frailty, frailty defined as a score below ten points [3]. GS is a quicker test, included in the SPPB. GS is individually validated to predict disability and all-cause mortality in community-dwelling older adults [14] and may be a valid, reliable, and sensitive tool for monitoring the functional status of older adults [15]. Screening tools are suggested as a first step in identifying and managing frailty. Further assessment and management, using Comprehensive Geriatric assessment (*CGA*), is recommended for older adults having an SPPB score below ten points or GS <0.8 m/s [10,11].

The prevalence of frailty is estimated to increase with the aging population. However, the condition represents a reinforced consequence of the aging process affected by lifestyle and comorbidity [1]. The condition and the sequelae may be prevented, reversed, or mitigated by tailored interventions [16]. Since the early stage of the condition may be symptomatically silent, it is proposed that early detection of frailty is essential to prevent or delay disabilities in home-dwelling older adults [4]. The optimal time point and the most suitable tool for frailty screening are under debate. The European consensus group, ADVANTAGE, has recommended opportunistic screening of everyone over 70 years, in all encounters with health care staff [11]. This is not practiced in Norway [17].

In Norway, National Insurance funded by taxes and grants from the state covers most health care costs [18]. Health promotion and public home care services are provided by the municipality. Home care services are either a safety alarm for older adults living alone and feeling unsafe, home cleaning assistance, assistive technology, physiotherapy interventions, home care reablement, or regular homecare. The citizen makes a formal inquiry, their needs are considered by a health professional, and services are assigned by given criteria. The first time an older adult applies for public home care service may therefore represent a feasible point for frailty screening.

The main purpose of the present study was to estimate the prevalence of frailty among older adults who apply for public home care services for the first time. The secondary purpose was to examine the appropriateness of GS and the SPPB as screening tools for frailty defined by Fried et al. [9] adapted to a general population >70 years in Norway.

## Methods

### Design

The present study had a cross-sectional design using data from a quality improvement project in Drammen in Norway, an urban municipality with 70,000 residents. The *Service assignment and coordinator office* assigned all older adults who applied for home care services for the first time, a functional assessment in their own home by a physiotherapist. The project period was from week 38/2018 to week 5/2019. The inclusion criteria were older adults >65 years who applied for home care services for the first time. Individuals who were not able to complete the tests were excluded from the study. Those with acute injuries, severe cognitive impairment, or an inability to communicate in the Norwegian language were excluded. Individuals with severe illnesses, for example, palliative cancer patients, were for ethical reasons not asked to participate in the project.

### Procedure and measurement tools

All assessments were completed in the participants’ homes by a trained physiotherapist. The following data were collected: self-reported age, sex, height, weight, and type of inquiry; (a) security alarm connected to health care, (b) home cleaning assistance, (c) assistive technology for movement or sight, (d) physiotherapy interventions/home care reablement, and (e) regular homecare. The FFP index was used to assess frailty. The components of the index were adapted to Norwegian settings. Details for assessment and cut points are presented in Table 1. The SPPB was assessed using the Norwegian version [19]. Unsupported standing balance was timed until the participant moved, or for ten seconds, in three positions: feet side-by-side, in semi-tandem, and tandem. The five times repeated chair-to-stand test was timed for participants who could perform this not supported by their hands. To assess GS, the participants were timed twice as they walked 4 m at their normal pace.

**Table 1.** Assessment tools and cut points for the five criteria in the FFP index, inspired by Fried et al. [9].

Criterium	Tool	Cut points
Weight loss	In the last year, have you lost weight unintentionally. What was your weight and what is your weight now?	Ye*s* > 5%
Exhaustion	Using the CES–D Depression Scale, the following two statements are read. (a) I felt that everything I did was an effort; (b) I could not get going. The question is asked ‘How often in the last week did you feel this way?’ 0: rarely or none of the time (<1 day), 1: some or a little of the time (1–2 days), 2: a moderate amount of the time (3–4 days), or 3: most of the time (>4 days).	Subjects answering ‘2’ or ‘3’ to either of these questions are categorized as frail by the exhaustion criterion
Physical activity	Based on the short version International Physical Activity Questionnaire. Kcals per week expended are calculated using standardized algorithms.	Kcals of physical activity per week <383 for men, kcals of physical activity per week <270 for women.
Usual gait speed	Assessed over 4 m inclusive acceleration	≤0.67 m/s for men ≤173 cm and women ≤159 cm,
		≤0.76 m/s for men ≥173 cm and women ≥159 cm
Grip strength	Average from 4 assessments with Handheld Jamar^®^ dynamometer	Depending on sex and BMI:
		Men; ≤29 kg if BMI ≤23, ≤30 kg if BMI 23.1–28, ≤32 if BMI >28.
		Women ≤17 if BMI ≤23, ≤17.3 if BMI 23.1–26, ≤18 kg if BMI 26.1–28, ≤21 kg if BMI >28.

### Statistical analyses

After a sample size calculation demanding confidence intervals (CI) at a maximum of 15% of a supposed prevalence of frailty at 40%, the study size arrived at a minimum of 116. Statistical analyses were performed by using IBM SPSS, version 25.0. We calculated proportions of categorical variables with 95% CI. The FFP index was used for categorisation of the participants. We defined frail as meeting three or more criteria, prefrail as meeting one or two criteria and robust as meeting no criteria for frailty. We calculated proportions of participants having GS <0.8 m/s and an SPPB-score below ten points. Means for ages, GS and SPPB-scores were presented with standard deviation (sd). We applied one-way ANOVA to investigate the association between chronological age and frailty status represented by frail, prefrail and robust groups, and to estimate statistically significant differences between both GS and SPPB-score and frailty status. We applied Chi trend tests to investigate differences between the frailty status groups in proportions having GS <0.8 m/s and proportions having an SPPB-score below ten points, respectively. A significance level of five percent was used in all statistical analyses. To examine the test accuracy values for GS and the SPPB to detect frailty, we defined *positive frail* as meeting three or more criteria for the FFP and *negative frail* as meeting two or fewer criteria. The prevalence of frailty in the present sample deviated significantly from the prevalence rate in a general population. We, therefore, used prevalence data for a general population >70 years in Norway reported by Langholz et al. [8], and the results within the *positive frail* category and the *negative frail* category *(prefrail and robust individuals)* from the present study. We calculated sensitivity, specificity, positive predicting value (PPV) and negative predicting value (NPV) as described by Fagerland and co-authors [20] for suggested cut point for GS at 0.8 m/s [10] and suggested cut point for SPPB-score below ten points [3]. We set the criteria for acceptable test accuracy to ≥80% sensitivity and ≥60% specificity, which reflect a standard of frailty screening in primary care [21]. Our data do not allow for calculating valid ROC-curves for a general population. To our knowledge, our procedure for calculating specificity and PPV does not allow for calculating CI.

### Ethics

All participants were informed orally and written and provided written consent before participating in the study. In the quality improvement project, all new appliers were offered an assessment. To ensure that participation in the study should not affect the offered health service, those who were assessed to be frail or prefrail were assigned appropriate health care before being asked for consent to participate in the study. The study was conducted in accordance with the Declaration of Helsinki [22] and approved by Regional Committees for Medical and Health Research Ethics (REC, reference 2018/1344) and Norwegian Centre for Research Data (NSD, reference 496235).

## Results

From a total of 129 older adults assessed in the quality improvement project, four did not consent to participate in the present study. Nine were excluded due to exclusion criteria, leaving 116 participants. Their age ranged from 65 to 93 years. Descriptive characteristics of the participants are presented in Table 2. 62.1% of the participants were assessed as frail, 29.3% as prefrail, and 8.6% as robust. GS ranged from 0.25 to 1.15 m/s, while the SPPB-scores ranged from one to 12.

**Table 2.** Characteristics of participants and results of assessment.

	Total	95% CI
Participants, *n*	116	
Men, *n*	43	
Women, *n*	73	
Age, mean ± sd	80.3 ± 6.7	
Type of inquiry		
Security alarm connected to health care service	47.4 %	[38.3%, 56.5%]
Practical cleaning assistance	16.4 %	[9.6%, 23.3%]
Assistive technology	48.3 %	[39.2%, 57.4%]
Physiotherapy interventions / home care reablement	24.1 %	[16.4%, 31.9%]
Homecare	31.0 %	[22.6%, 39.5%]
Criteria for Frieds’ Frailty Phenotype		
Un-intentional weight loss	35.3 %	[26.6%, 44.0%]
Self-reported exhaustion,	54.3 %	[45.2%, 63.4%]
Low physical activity	50.0 %	[40.9%, 59.1%]
Slowness	70.7 %	[62.4%, 79.0%]
Weakness (low grip strength)	61.2 %	[52.3%, 70.0%]
Frailty status		
Frail, meeting 3 or more criteria for frailty	62.1 %	[53.3%, 70.9%]
Prefrail, meeting 1 or 2 more criteria for frailty	29.3 %	[21.0%, 37.3%]
Robust, meeting no criteria for frailty	8.6 %	[3.5%, 13.7%]
GS (m/s), mean ± sd	0.66 ± 0.19	
GS < 0.8 m/s	87.1 %	[81.0%, 91.2%]
SPPB-score, mean ± sd	7.8 ± 2.6	
SPPB-score ≤ 9	68.1 %	[59.6%, 76.6%]

Table 3 presents age, GS and SPPB-scores in the frail, prefrail and robust groups. There was no statistically significant association between age and frailty (*p* = 0.626). Mean GS and SPPB-scores were significantly lower in the frail group than in the prefrail group and significantly lower in the prefrail group than in the robust group (*p* < 0.001). These differences were considered clinically significant [13,14]. None in the robust group had GS under <0.8 m/s or an SPPB-score below ten points.

**Table 3.** Age, GS and SPPB-scores in frail, prefrail and robust group.

	Frail (*n* = 72)	Prefrail (*n* = 34)	Robust (*n* = 10)	*p*-Value
Mean age ± sd	78.7 ± 6.0	80.1 ± 6.9	80.6 ± 6.6	0.626^a^
Mean GS (m/s)±sd	0.58 ± 0.13	0.75 ± 0.15	0.99 ± 0.13	**<0.001^a^**
GS < 0.8 m/s, *n* (%)	71 (98.7)	27 (80.0)	0	**<0.001^b^**
Mean SPPB-score ± sd	6.8 ± 2.3	8.9 ± 1.9	11.5 ± 0.7	**<0.002^a^**
SPPB score ≤ 9, *n* (%)	61 (84.7)	18 (54.5)	0	**<0.001^b^**

^a^One-way ANOVA; ^b^Chi trend test. Bold values are statistically significant *p*-values.

Test accuracy values for GS <0.8 m/s and a SPPB-score below ten points to detect frailty are presented in Table 4.

**Table 4.** Test accuracy values for GS <0.8 m/s, SPPB below nine points to detect frailty (Three or more criteria of the PFF index).

	Sensitivity	Specificity	Positive predictive value	Negative predictive value
GS < 0.8 m/s	0.99	0.68	0.11	1.00
SPPB ≤ 9	0.85	0.78	0.13	0.99

## Discussion

Among Norwegian older adults >65 years applying for public home care services for the first time, we found that the majority (62.1%) were frail and that 29.3% were prefrail according to the FFP index. We did not find other studies reporting the prevalence rate for frailty in this population in Norway or in other countries. A few studies have investigated the prevalence of frailty among older adults receiving home care in Europe, showing prevalence rates between 24–75% [23]. The present study indicates that most home-dwelling older adults in Norway do not ask for minor public home care services, that is, security alarm, home care reablement, or assistive technology before they have developed frailty, despite low costs or being free of charge. However, there was no association between chronological age and frailty in this population, as is found for a general population [7]. It is thereby reasonable to assume that the request for public home care services may appear as a result of frailty and not as a result of ageing. This is supported by the theoretical concept of frailty as an indication of a person’s ‘biological age’ rather than chronological age [1,24].

International consortiums propose that screening with a simple instrument represents the first step in the identification and management of frailty and should be followed up by a diagnostic tool and a CGA [4,10,11]. Interventions based on a CGA may increase the patient’s intrinsic capacity and quality of life, prevent disability, slow down the progression of frailty, and lessen the burden on next of kin [25]. Based on the high prevalence of frailty in the present study, one can argue that older adults should be assessed for frailty when they apply for public home care services for the first time in Norway. This is not the practice today [17]. On the other hand, to prevent frailty and intervene when improvement is still possible, the present study indicates that older adults should be screened for frailty at an *earlier time point*. This is supported by international consortiums proposing screening of all older adults over 70 years in all meetings with health and social staff [11,26]. The screening tool, however, should be quick to administer, non-invasive and gentle, not require special equipment and be validated as well as being sensitive, specific and have good predictive values [11]. Both GS and the SPPB represent quick, simple, and low-cost assessment tools, feasible in ordinary health care offices or in ordinary homes.

The relevance of investigating test accuracy values for GS or SPPB-scores to detect frailty defined by Fried et al. [9] may be questioned as slowness is one of the five criteria in the FFP index. However, according to Clegg et al. [10], it is of importance to find valid, easy-to-use screening tools for clinical settings [10,11]. GS may represent a quick, safe, and low-cost tool. It can be measured in under a minute, the SPPB takes 5 min, while measuring the FFP takes over 15 min and requires special equipment.

In this study, both mean GS and mean SPPB-scores were significantly lower in frail compared to prefrail individuals and significantly lower in prefrail compared to robust individuals. Sensitivity, specificity, and NPV by using GS <0.8 m/s and an SPPB-score below ten points to detect frailty were all beyond the standard for acceptable test accuracy values at over 80% sensitivity and over 60% specificity [21]. However, the present study indicates that GS at a cut point of 0.8 m/s may be more appropriate than the SPPB as a screening tool to detect frailty in a general population >70 years in Norway. Our test accuracy results for GS <0.8 m/s and the test accuracy results for a general population >65 years in Spain were similar [27]. A study on general practice patients aged >75 years in South Australia reported lower sensitivity (70%), though acceptable specificity (77%) and PPV (39%) on GS ≤0.8 m/s to detect frailty [21]. There are other, modified validated screening tools for frailty [3,11]. It is proposed that the screening tool should be chosen according to context, population characteristics, and aim [4]. Assessing GS does not have language barriers and is familiar to health professionals in Norway [12]. There is increasing evidence that GS alone may be considered as a valid, reliable, and sensitive tool for monitoring functional status and overall health for older adults [15]. Underlying causes of slowness may also be related to the impairment of multiple physiological systems, a description that corresponds with the overall understanding of frailty [28].

The PPV for GS was 11%, which may indicate weakness of a screening tool. However, PPV is dependent on the prevalence rate and is typically low in rare medical conditions [21]. PPV in the present study is comparable to that of the mammography screening program in Norway [29]. When calculating test accuracy values for GS and SPPB-scores to detect frailty, the participants assessed as prefrail were classified as *negative frail*. Sensitivity and specificity are rarely dependent on prevalence in the target group [21]. However, the prevalence of prefrailty among those classified as *negative frail* influences both specificity and PPV in our calculation as low GS often is common also in persons assessed, as prefrail. 80% in the prefrail group had GS <0.8 m/s and 54.5% had an SPPB-score below ten points. In comparison, none in the robust group had GS <0.8 m/s nor an SPPB-score below 10 points. Bandeen-Roche et al.(2019) proposed the need to detect prefrailty and to improve prefrailty measures, in order to intervene when improvement is still possible [30]. The present study has a too-small sample size in order to provide valid results for the subgroup of robust individuals and to calculate test accuracy values of GS and SPPB-scores that can differentiate between prefrail and robust individuals. Research on larger selections is needed to investigate this.

The present study was conducted in a medium-sized municipality in Norway. There are significant differences in healthcare systems between countries. Norwegian citizens have relatively easy access to public health care to low deductibles, compared to other countries. The generality of the results may be limited to Norwegian conditions or similar countries. The open definition of frailty, and lack of international consensus, also represent a limitation in comparing prevalence studies [23]. Our procedure for calculating test accuracy values for GS and SPPB-scores might be questioned. However, it is challenging to develop a screening tool, with acceptable and valid PPV based on small sample sizes. It was not appropriate to provide valid test accuracy values based on the distribution of frailty in our study, as the prevalence deviated significantly from general populations. We, therefore, chose to use the prevalence data from a study with larger sample size and a normal distribution of frailty. We considered the 5 years difference in age limit to be acceptable, as only a few participants in our study were below 70 years. The present study did not examine socio-demographic and socio-economic characteristics. This is a limitation of the study.

## Conclusion

In conclusion, the present study has generated novel and valuable knowledge about the prevalence of frailty in a group of older adults at the point they seek home care services in Norway. The high prevalence of frailty in this population indicates that an earlier point for frailty screening may be beneficial. The ideal setting and time for frailty screening programs for older adults should be further investigated. The present study indicates that GS at a cut point at 0.8 m/s may be a feasible and easy-to-use screening tool for frailty, calculated for a general population >70 years in Norway.
